# 2486. Winners and Losers: MDROs In The Time of COVID-19

**DOI:** 10.1093/ofid/ofad500.2104

**Published:** 2023-11-27

**Authors:** Hwang Ching Chan, Geraldine Tingting Foo, Ka Lip Chew, Paul Ananth Tambyah, Jyoti Somani

**Affiliations:** National University Hospital, Singapore, Not Applicable, Singapore; National University Hospital, Singapore, Not Applicable, Singapore; National University Hospital, Singapore, Singapore, Not Applicable, Singapore; Department of Medicine, Infectious Diseases, National University Hospital, Singapore, Western Area, Singapore; National University Hospital, Singapore, Not Applicable, Singapore

## Abstract

**Background:**

NUH is a 1200 bed academic medical center in Singapore, with endemic multi-drug resistant infections (MDROs) including carbapenemase-producing Enterobacterales (CPE), vancomycin-resistant Enterococci (VRE), Clostridioides difficile infection (CDI), and multi-drug resistant(MDR) Acinetobacter spp. (AB), and MDR Pseudomonas aeruginosa (PA). We studied the impact of COVID-19 (C19) restrictions on the prevalence of hospital acquired (HA) MDROs.

**Methods:**

A HA infection is defined as a positive clinical culture after hospital day 3. The rates of key MDROs were reviewed from 2018 to the start of the zero C19 policy in Feb 2020 through the lifting of zero COVID in Aug 2021 (Delta Surge) with services still diverted until Jan 2022, when the hospital policy shifted to Endemic C19 (all clinical services resumed). Incidence rates (IR) are expressed per 10,000 patient days, and incidence rate ratios (IRR) estimated for each time period, with p< 0.05 considered statistically significant. We compared DDD per 100 patient days of carbapenem usage for each time period.

**Table 1:**
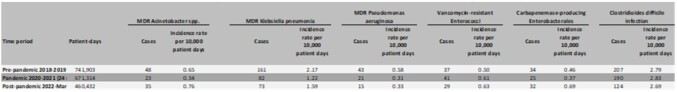
Incidence rates of HO CPE, VRE, CDI, and MDR AS, KP, PA, comparing pre-pandemic 2018-2019, pandemic 2020-2021, and post-pandemic 2022-March 2023

**Table 2:**
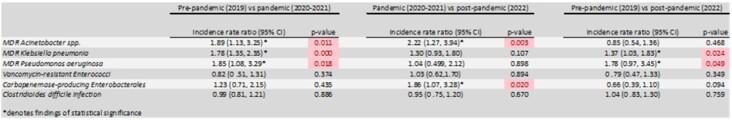
Incidence rate ratios of HO CPE, VRE, CDI, and MDR AS, KP, PA, comparing pre-pandemic 2018-2019, Zero-COVID 2020 July 2021, Delta surge Aug- Dec 2021-and Endemic COVID Jan 2022- 2023

**Results:**

While HA-MDRAB rates declined during the zero C19 period, VRE and CDI rates were stable. CPE rates decreased during the pandemic but have almost doubled post-pandemic from 0.37 in the pandemic to 0.69 post-pandemic (p< 0.05). The mean DDD per 100 patient days for carbapenem usage pre-pandemic was 5.24 and fell to 4.64 (p< 0.05) in 2020-2021.

**Figure 3:**
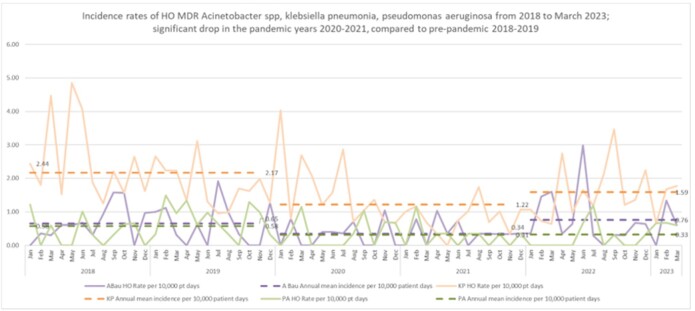
Incidence rates of MDROs that significantly trended downwards during the zero COVID and the Delta surge periods.

**Figure 4:**
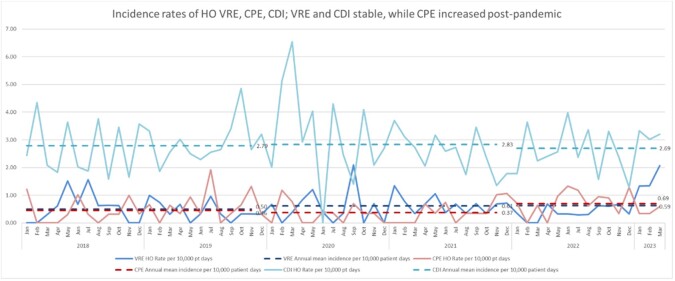
Incidence rates of VRE and CDI appeared stable, while CPE increased post-pandemic 2022-March 2023

**Figure 5:**
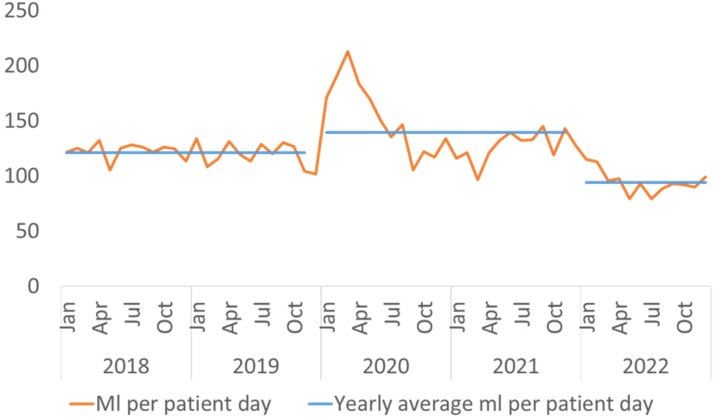
Procurement of alcohol-based hand rub pre-pandemic, during pandemic, and post-pandemic

**Conclusion:**

Studies globally report increased MDROs during the pandemic, purportedly due to reuse of PPE and other reasons. We experienced either a significant decrease or no change in many MDRO clinical infections, followed by a rebound since Jan 2022, when all hospital services resumed (Endemic C19). Our decrease in MDROs may be due to our zero C19 policy, increased hand hygiene, enhanced PPE adherence and cleaning regimens, a decrease in our carbapenem usage (p< 0.05 Table 6), and reduced elective surgeries and admissions, including overseas patients . After endemicity, we had a rebound in CPE infections, and other MDROs possibly triggered by HH and PPE fatigue (Fig. 5), the return of medical tourists and elective surgeries. Further prospective studies are needed to better understand the epidemiology of the various MDROs and the impact of draconian measures such as the Asian Zero C19 approach.

**Table 6:**
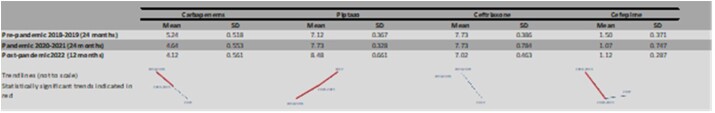
Defined daily dose of antibiotics per 100 patient days (by quarter)

**Disclosures:**

**All Authors**: No reported disclosures

